# Feasibility of Embedding a Scalable, Virtually Enabled Biorepository in the Electronic Health Record for Precision Medicine

**DOI:** 10.1001/jamanetworkopen.2020.37739

**Published:** 2021-02-22

**Authors:** Kimberley M. DeMerle, Jason N. Kennedy, Octavia M. Peck Palmer, Emily Brant, Chung-Chou H. Chang, Robert P. Dickson, David T. Huang, Derek C. Angus, Christopher W. Seymour

**Affiliations:** 1 The Clinical Research, Investigation, and Systems Modeling of Acute illness Center, Department of Critical Care Medicine, University of Pittsburgh School of Medicine, Pittsburgh, Pennsylvania; 2 Division of Pulmonary, Allergy, and Critical Care Medicine, University of Pittsburgh, Pittsburgh, Pennsylvania; 3 Department of Critical Care Medicine, University of Pittsburgh, Pittsburgh, Pennsylvania; 4 Department of Pathology, University of Pittsburgh, Pittsburgh, Pennsylvania; 5 Department of Medicine, University of Pittsburgh, Pittsburgh, Pennsylvania; 6 Division of Pulmonary and Critical Care Medicine, Department of Medicine, University of Michigan, Ann Arbor; 7 Department of Microbiology & Immunology, University of Michigan Medical School, Ann Arbor; 8 Office of Healthcare Innovation, University of Pittsburgh Medicine Center Health System, Pittsburgh, Pennsylvania; 9 Senior Editor, *JAMA*; 10 Department of Emergency Medicine, University of Pittsburgh, Pittsburgh, Pennsylvania; 11 Associate Editor, *JAMA*

## Abstract

**Importance:**

A cornerstone of precision medicine is the identification and use of biomarkers that help subtype patients for targeted treatment. Such an approach requires the development and subsequent interrogation of large-scale biobanks linked to well-annotated clinical data. Traditional means of creating these data-linked biobanks are costly and lengthy, especially in acute conditions that require time-sensitive clinical data and biospecimens.

**Objectives:**

To develop a virtually enabled biorepository and electronic health record (EHR)–embedded, scalable cohort for precision medicine (VESPRE) and compare the feasibility, enrollment, and costs of VESPRE with those of a traditional study design in acute care.

**Design, Setting, and Participants:**

In a prospective cohort study, the EHR-embedded screening alert was generated for 3428 patients, and 2199 patients (64%) were eligible and screened. Of these, 1027 patients (30%) were enrolled. VESPRE was developed for regulatory compliance, feasibility, internal validity, and cost in a prospective cohort of 1027 patients (aged ≥18 years) with sepsis-3 within 6 hours of presentation to the emergency department. The VESPRE infrastructure included (1) automated EHR screening, (2) remnant blood collection for creation of a virtually enabled biorepository, and (3) automated clinical data abstraction. The study was conducted at an academic institution in southwestern Pennsylvania from October 17, 2017, to June 6, 2019.

**Main Outcomes and Measures:**

Regulatory compliance, enrollment, internal validity of automated screening, biorepository acquisition, and costs.

**Results:**

Of the 1027 patients enrolled in the study, 549 were included in the proof-of-concept analysis (305 [56%] men); median (SD) age was 59 (17) years. VESPRE collected 12 963 remnant blood and urine samples and demonstrated adequate feasibility for clinical, biomarker, and microbiome analyses. Over the 20-month test, the total cost beyond the existing operations infrastructure was $39 417.50 ($14 880.00 project management, $22 717.50 laboratory supplies/staff, and $1820.00 data management)—approximately $39 per enrolled patient vs $239 per patient for a traditional cohort study.

**Conclusions and Relevance:**

Results of this study suggest that, in a large US health system that collects data using a common EHR platform and centralized laboratory system, VESPRE, a large-scale, inexpensive EHR-embedded infrastructure for precision medicine can be used. Tested in the sepsis setting, VESPRE appeared to capture a high proportion of eligible patients at low incremental cost.

## Key Points

**Question:**

Is use of a large-scale, inexpensive electronic health record–embedded infrastructure that captures a high proportion of eligible participants and creates a virtually enabled clinical and biologic repository feasible?

**Findings:**

In this cohort study of 1027 patients with sepsis, a novel infrastructure, termed virtually enabled biorepository and electronic health record–embedded, scalable cohort for precision medicine (VESPRE) was developed. VESPRE appeared to demonstrate feasible digital screening, successful enrollment, biologic sampling, and lower costs compared with a traditional study design.

**Meaning:**

Results of this study suggest that VESPRE, an infrastructure embedded in the EHR that creates a virtually enabled biorepository, may be scalable across patients, centers, and laboratories at low incremental cost and burden.

## Teaser

This cohort study examines a virtually enabled biorepository and electronic health record (EHR)–embedded tool for use in precision medicine in patients with sepsis.

## Introduction

Clinicians have increasingly shifted from a treatment approach used for all patients to one that targets the individual.^1^ Informed by clinical, genomic, proteomic, or other tissue markers, clinicians treat patients with breast cancer, melanoma, and asthma, among other diagnoses, with life-saving precision care.^2,3^ However, progress toward precision medicine has extended less quickly to acute care. Major barriers are the lack of large-scale and adequately detailed data on many disease states and phenotypes, as well as a narrow time window to collect clinical and biological data relevant to time-sensitive treatment,

There are many challenges to precision medicine in acute, time-sensitive conditions. Traditional prospective cohort studies, constructed as stand-alone research initiatives, can be lengthy and costly. Studies with conventional designs using manual screening and informed consent may enroll only a small proportion of eligible patients. These steps limit sample size and generalizability. Traditional prospective cohort studies also require costly manual data collection with auditing, which limit data sets to prespecified groups of clinical and biological variables. In addition, many acute illnesses are difficult to recognize during early care, challenging the rapid deployment of inclusion criteria. A new strategy is needed to create a low-burden, rich-knowledge network with adequate internal validity while accounting for diagnostic uncertainty.^4,5,6,7,8,9^

To approach these challenges, the National Institutes of Health funded a digital strategy termed VESPRE (virtually enabled biorepository and electronic health record [EHR]–embedded, scalable cohort for precision medicine). VESPRE is a low-burden infrastructure that uses automated EHR algorithms to prospectively screen and enroll eligible patients with regulatory approval to create a biorepository of clinical data and remnant biologic samples. We tested VESPRE in sepsis, a life-threatening condition that accounts for 1 in 3 hospital deaths.^10,11,12^ Sepsis is heterogeneous, vague in its clinical presentation, and the focus of many precision medicine initiatives.^1,4,7^ We describe the development and implementation of a VESPRE in sepsis, and provide proof of concept for digital screening, enrollment, biologic sampling, preliminary analyses, and costs compared with a traditional study design.

## Methods

**Figure 1.**
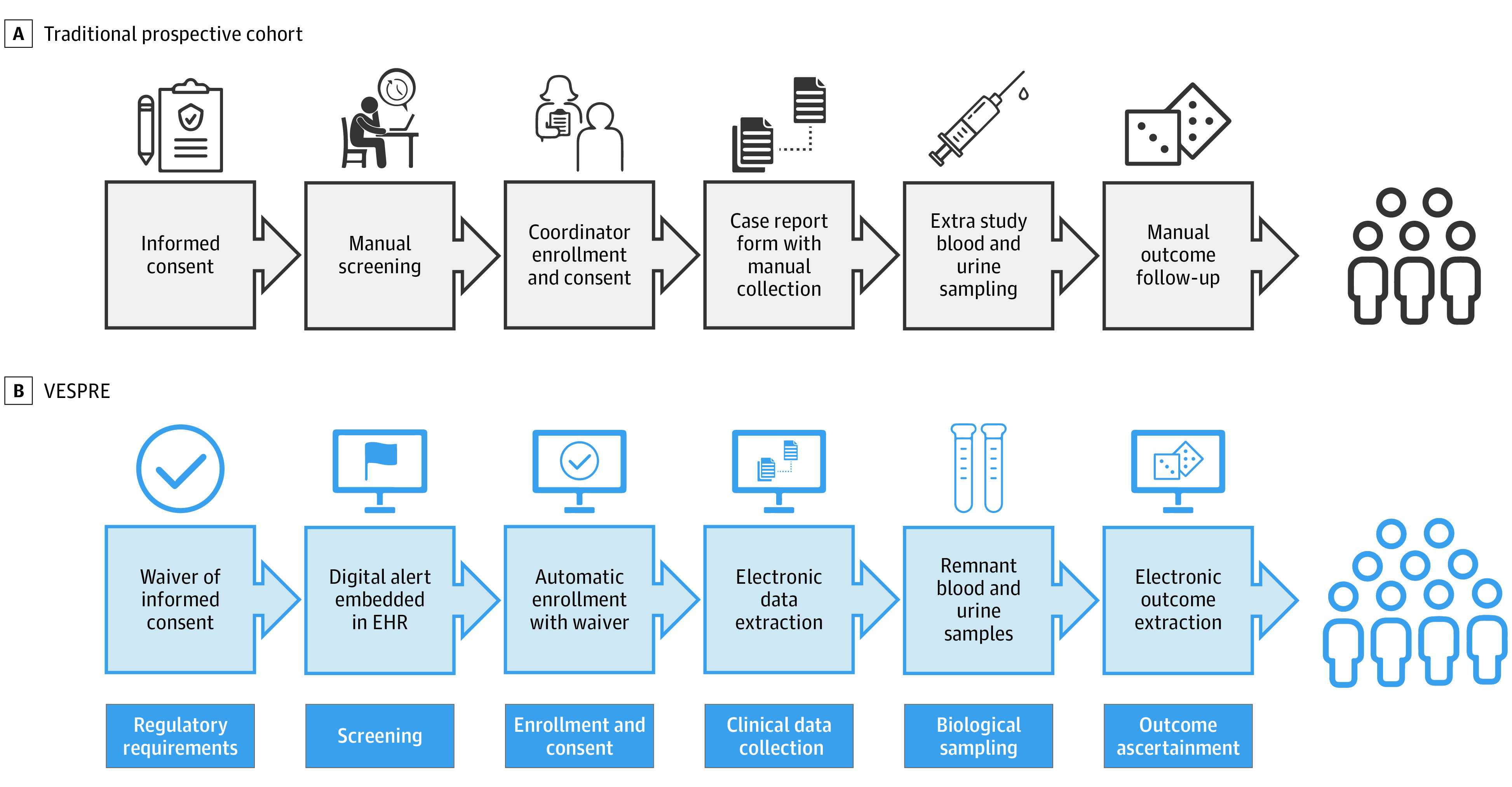
Study Design Contrasting the study design of a traditional prospective cohort study (A) and virtually enabled biorepository and electronic health record (EHR)–embedded, scalable cohort for precision medicine (VESPRE) (B), categorized by key study design steps.

The 3 goals for VESPRE were to (1) create a digital tool for patient screening and enrollment embedded in the EHR; (2) develop automated sample collection for a virtually enabled biorepository of waste tissue, defined as remnant blood and urine specimens; and (3) develop a thin-client software that ensures adequate data integration, security, curation, and scalability across multiple hospitals. In this approach, we combined 3 components (existing tools of EHR sepsis screening, EHR data for clinical research, and remnant sample analyses) to accomplish these goals. A summary of VESPRE study design, compared with a traditional prospective cohort study, is detailed in Figure 1. This study followed the Strengthening the Reporting of Observational Studies in Epidemiology (STROBE) reporting guideline. The project was considered under expedited, full committee review by the University of Pittsburgh Institutional Review Board. VESPRE was approved with a waiver of informed consent and authorization under the Health Insurance Portability and Accountability Act.

### Regulatory Process and Data Collection

A key design feature was to seek a waiver of consent for VESPRE, reduce time and cost, and allow rapid enrollment with minimal patient risk. Patient screening, enrollment, and sample acquisition were completed prospectively. Daily screening alerts were generated through a secure communication that reported potentially eligible encounters to the study team. The study team verified eligibility and collected remnant blood samples for indicated patients. Patient identifiers were removed and replaced with study identification numbers immediately after collection and before storage in the biorepository. Deidentified data were used in the analysis, which was completed in a batched format. A study code linking the deidentified data with patient identifiers was maintained separately. These deidentified samples linked to data were abstracted from Cerner (Cerner Powerchart; Cerner) in bulk files onto SQL Server and stored securely behind existing University of Pittsburgh Medicine Center (UPMC) firewalls. Only research staff who completed training had access to the master crosswalk to prevent improper use or disclosure. Oversight was provided by Clinical Research, Investigation, and Systems Modeling of Acute Illness Center Biostatistics and Data Management Core and the University of Pittsburgh. Prespecified variables were outlined before the study. Electronic health record data included patient demographics; physiologic and laboratory parameters, including those used for the Sequential Organ Failure Assessment (SOFA) score; and interventions (eg, provision of mechanical ventilation, medication administration, and short- and long-term outcomes). The data collected were intensive care unit admission, hospital and intensive care unit length of stay, in-hospital mortality, and 90-day mortality.

### Study Population and Setting

VESPRE was tested in a prospective cohort in southwestern Pennsylvania at UPMC Presbyterian from October 17, 2017, to June 6, 2019. Eligible patients included all adults (aged ≥18 years) who met digitally obtained criteria for sepsis within 6 hours of presentation to the emergency department. Sepsis was defined as sepsis-3, including evidence of a suspected infection and presence of acute organ dysfunction.^13^ Sample size was determined by available funding for this feasibility pilot study.

### Digital Screening and Enrollment Using the EHR

**Figure 2.**
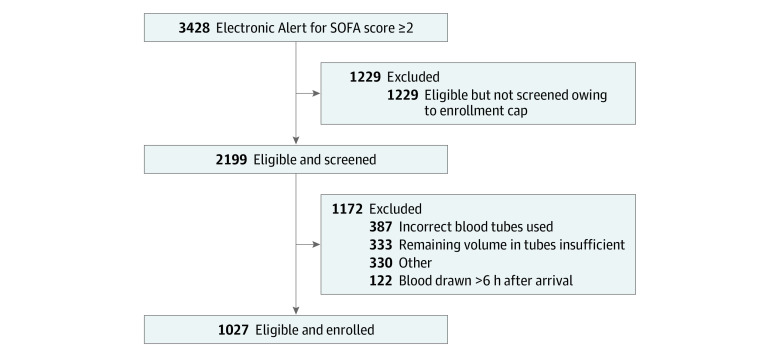
Patient Screening and Enrollment SOFA indicates Sequential Organ Failure Assessment.

To identify sepsis-3 within 6 hours of presentation, we developed a digital alert to flag patients with (1) location in the enrolling emergency department, (2) age 18 years or older, (3) blood cultures obtained in the first 6 hours after arrival, (4) antibiotics administered in the first 6 hours, (5) 1 of 6 modified SOFA elements^14^: Pao_2_/Fio_2_ less than 400 mm Hg, Glasgow Coma Scale score lower than 15, mean arterial pressure less than 70 mm Hg or vasopressors administered, total bilirubin greater than 1.2 mg/dL (to convert to micromoles per liter, multiply by 17.104), platelets less than 150 × 10^3^/μL (to convert to ×10^9^ per liter, multiply by 1), creatinine greater than 1.2 mg/dL (to convert to micromoles per liter, multiply by 88.4), and (6) hemoglobin, plasma lactate, and sodium levels obtained. These laboratory tests were required to ensure the proper tube types were available for current and future VESPRE analyses. When a digital screening alert occurred, VESPRE staff were electronically notified to coordinate the collection of remnant samples. Flagged patients were categorized as (1) eligible and not screened owing to a daily enrollment cap of 4 patients, (2) eligible but excluded, and (3) enrolled. Flagged patients were eligible but excluded if (1) specimens were not collected within the 6-hour window, (2) volume in the remnant tubes was insufficient, or (3) the minimum number of required tube types was absent (Figure 2). Manual clinical adjudication of the modified SOFA score alert was completed by 4 blinded reviewers (including K.M.D. and J.N.K.) to determine the scores in a random sample of 50 patients.

### Remnant Samples

We collected remnant blood and urine samples in the VESPRE biorepository to maximize the number of samples and minimize time and cost. The biorepository is considered virtually enabled in that the embedded electronic alert triggers research staff to collect remnant samples without study team intervention. All remnant blood and urine samples were processed for their original collection purpose according to standard clinical laboratory practices. Staff then obtained remnant specimens from the clinical laboratory within 48 hours of acquisition, after clinical testing was complete, and before discard. The minimal remaining heparin (>550 μL), EDTA (≥450 μL), and sodium fluoride (≥225 μL) blood specimens were assessed. Although urine samples were not necessary for study enrollment, the minimum remaining urine volume of any available samples was 125 μL. Remnant blood and urine samples were transported to the research laboratory and processed on the same day that they were obtained from the clinical laboratory. Samples were aliquoted and stored at −80 °C in the VESPRE biorepository.

To demonstrate the feasibility of using remnant samples for analyses, specimens were tested for biomarkers contributing to potential sepsis phenotypes: plasminogen activator inhibitor-1, antithrombin III, interleukin-6, interleukin-8, interleukin-10, E-selectin, angiopoietin 1, angiopoietin 2, intracellular adhesion molecule, heme-oxygenase 1, tissue inhibitor of metalloproteinases 2, insulinlike growth factor binding protein 7, lactate, bicarbonate, uric acid, C-reactive protein, and procalcitonin.^7^ Details on test type, minimal sample volume, and sample tube type are provided in eTable 1 in the Supplement.

To determine the feasibility of remnant blood for analysis of bacterial dissemination (attributable to bacteremia or translocation of gut bacteria), the samples underwent DNA extraction, bacterial DNA quantification, and 16S rRNA gene amplicon sequencing. Genomic DNA was assessed (Qiagen DNeasy Blood & Tissue kit, Qiagen). Numerous negative controls were included to discriminate bacterial signal from sequencing contamination: elution buffer (n = 6), extraction controls (n = 7), sterile water (n = 3), and blank wells (n = 30). Sequencing was done via MiSeq Illumina and quantification was done using droplet digital polymerase chain reaction.^15,16^ We performed microbial ecology analysis using the *vegan* package 2.5-5 and *mvabund* in R, version 3.6.1 as previously described.^17^

### Assessments of Study Costs

To estimate the financial implications of VESPRE for patient screening, enrollment, and biologic sampling, the cost of VESPRE was compared with a prehospital cohort study^18^ and a hypothetical prospective cohort among hospitalized patients. The Pittsburgh Prehospital Linking Evaluation is a National Institutes of Health–funded prospective cohort study of prehospital patients that integrated emergency medical service records and hospital-based EHRs with coordinator-collected blood samples.^18,19^ The hypothetical prospective cohort model was calculated based on the estimated costs for a research coordinator core to complete the same incremental activities for VESPRE for 1027 enrolled patients, including obtaining informed consent and collecting fresh blood and urine samples. Costs are expressed as total cost and cost per patient enrolled and separated as project management, laboratory supplies and staff, and data management.

### Statistical Analysis

To assess the feasibility of VESPRE for patient enrollment and virtual biorepository creation, heat maps and summary statistics for patients, protein biomarkers, and microbiome were generated. Summary statistics are presented as mean (SD) or median (interquartile range), as appropriate.

To examine the feasibility of VESPRE data for machine learning in precision medicine, we applied factor analysis and generated reachability plots to candidate clinical variables. Factor analysis was completed on 29 clinical variables that were collected within 6 hours of presentation to the emergency department. Factor analysis is a statistical technique that aims to find similar patterns (unobserved factors) among multiple observed variables within a data set.^20^ In addition, to assess the optimal clustering method for the data, we applied a density-based clustering algorithm (ie, ordering points) to identify the clustering structure (OPTICS).^21^ OPTICS detects natural clusters with various densities and generates a reachability plot, which provides an overall visualization of data structure to guide the choice of the optimal clustering method of the data set. If the reachability plot is jagged (representing relatively clear natural cluster structure in the data), hierarchical clustering is recommended. In contrast, if the reachability plot is smooth, partitioning methods are recommended. For comparisons, we used χ^2^ test for categorical data. The threshold for statistical significance was *P* < .05 for 2-sided tests. Data analysis was conducted using Stata, version 16.1 (StataCorp LLC) and R software, version 3.6.2 (R Foundation for Statistical Computing).

## Results

### Regulatory Review and Study Procedures

For the waiver of informed consent, the University of Pittsburgh Institutional Review Board stipulated that use of clinical data from the EHR and remnant blood and urine samples presented no more than minimal risk to the patients. To ensure the protocol could meet minimal risk, we did not collect, store, or process any genetic material. Remnant blood and urine samples were collected up to 48 hours after sampling, after clinical testing was complete, and before discard. All data were stored securely behind existing health system firewalls, linked by a master crosswalk with protected health information.

### Automated Alerting and Enrollment

Among 42 893 emergency department encounters during the 20-month study period (October 17, 2017, to June 1, 2019), 3428 patients were flagged with a VESPRE digital alert. A total of 2199 patients (64%) were eligible and screened; 1229 patients were not screened because of the enrollment cap (n = 4), and 1027 of the eligible patients were enrolled (Figure 2; eFigure 1 in the Supplement). Patients who received a SOFA alert but were excluded included 1175 individuals (34%) with incorrect specimen tubes used (33%) or insufficient specimen volume (28%) (eFigure 2 in the Supplement). Among the 1027 enrolled patients, clinical data were evaluated in proof-of-concept analyses (n = 549). The median (SD) age of the patients was 59 (17) years and the mean (SD) number of Elixhauser comorbidities was 4.0 (2.3) (Table). A total of 305 patients (56%) were male and 244 were female (44%). Nearly half of the patients (249 [45%]) were admitted to the intensive care unit and 1 in 6 required vasopressors (78 [14%]). Inpatient mortality was 9% (n = 49), and 90-day mortality was 17% (n = 94). Manual adjudication revealed that the SOFA score was greater than or equal to 2 in all 50 patients evaluated. On average, missingness of clinical data was low (<25% for 23 of 29 variables) (eTable 2 in the Supplement).

**Table.** Patient Characteristics in Nested Sample

Variable	Total (n = 549)^a^
Demographic	
Age, mean (SD), y	59 (17)
Sex, No. (%)	
Male	305 (56)
Female	244 (44)
Race, No. (%)^b^	
White	364 (83)
Black	69 (16)
Other	8 (2)
Elixhauser comorbidities, mean (SD), No.	4.0 (2.3)
Inflammation	
Premature neutrophil count (bands), median [IQR], %	10.0 [4.0-21.0]
C-reactive protein, mean (SD), mg/L	12.5 (12.1)
Erythrocyte sedimentation rate, mean (SD), mm/h	46.0 (52.3)
White blood cell count, mean (SD), ×10^9^/L	13.4 (8.8)
Pulmonary	
Respiratory rate, mean (SD), breaths/min	23.6 (6.5)
Oxygen saturation, mean (SD), %	92.9 (5.3)
Partial pressure of oxygen, mean (SD), mm Hg	134.3 (74.1)
Cardiovascular or hemodynamic	
Heart rate, mean (SD), beats/min	104.8 (22.5)
Systolic blood pressure, mean (SD), mm Hg	104.9 (24.3)
Troponin I, median [IQR], ng/mL	0.1 [0.1-0.2]
Serum lactate, median [IQR], mmol/L	1.4 [1.0-2.2]
Bicarbonate, mean (SD), mEq/L	22.9 (4.7)
Renal	
Creatinine, median [IQR], mg/dL	1.4 [1.0-2.3]
Blood urea nitrogen, median [IQR], mg/dL	25 [16-41]
Hepatic	
Albumin, mean (SD), g/dL	3.3 (0.7)
Alanine aminotransferase, median [IQR], U/L	21 [12-42]
Aspartate aminotransferase, median [IQR], U/L	29 [17-52]
Bilirubin, median [IQR], mg/dL	0.8 [0.5-1.5]
Hematologic	
Hemoglobin, mean (SD), g/dL	10.9 (2.3)
International normalized ratio, mean (SD)	1.5 (0.9)
Platelet count, median [IQR], ×10^3^/μL	208 [144-278]
Other	
Chloride, mean (SD), mEq/L	101.6 (5.7)
Glucose, median [IQR], mg/dL	129 [104-171]
Sodium, mean (SD), mEq/L	135.3 (7.3)
Glasgow Coma Scale score, mean (SD)	11.8 (4.3)
Outcomes, No. (%)	
Intensive care unit admission	249 (45)
Mechanical ventilation	108 (20)
Vasopressors	78 (14)
Inpatient mortality	49 (9)
90-d mortality	94 (17)

Abbreviation: IQR, interquartile range.

SI conversion: To convert alanine aminotransferase to microkatals per liter, multiply by 0.0167; albumin to grams per liter, multiply by 10; aspartate aminotransferase to microkatals per liter, multiply by 0.0167; bicarbonate to millimoles per liter, multiply by 10; bilirubin to micromoles per liter, multiply by 17.104; blood urea nitrogen to millimoles per liter, multiply by 0.357; chloride to millimoles per liter, multiply by 1; C-reactive protein to milligrams per liter, multiply by 10; creatinine to micromoles per liter, multiply by 88.4; glucose to millimoles per liter, multiply by 0.0555; hemoglobin to grams per liter, multiply by 10; lactate to millimoles per liter, multiply by 0.111; neutrophil bands to proportion of 1.0, multiply by 0.01; platelets to ×10^9^ per liter, multiply by 1; sodium to millimoles per liter, multiply by 1; troponin I to micrograms per liter, multiply by 1; and white blood cell count to ×10^9^ per liter, multiply by 0.001.

^a^ Variables measured within the first 6 hours of emergency department arrival. Outcomes measured as binary outcomes during inpatient hospitalization.

^b^ Data available on 441 patients.

### Virtually Enabled Biorepository

A total of 3 054 644 specimens were collected in the clinical laboratory during the study period. Of these, 12 963 remnant blood and urine samples (0.4%) were identified from VESPRE patients. A total of 1021 patients (99%) had 2 or more aliquots of any type available for study. Most patients had 2 or more aliquots of the sample tube type available for testing (citrate, 674 [66%]; EDTA, 463 [45%]; heparin, 518 [50%]; and sodium fluoride, 581 [57%]). A total of 1632 remnant urine samples were identified in VESPRE patients. One-third of the patients (365 [35%]) had at least 2 urine samples available for testing. Of the eligible patients, only 333 (28%) were excluded owing to insufficient specimen volume (eFigure 2 in the Supplement). The remnant blood and urine samples were stored indefinitely in one 21 cubic foot freezer, purchased for $13 035.00.

### Proof-of-Concept Analyses

Clinical data from VESPRE were used in preliminary machine learning analyses. A factor analysis of 23 clinical variables (6 clinical variables excluded owing to missingness) revealed 5 factors that explained most of the variability in the data (eigenvalue >1) (eFigure 3 in the Supplement). All 5 factors loaded on clinically meaningful variables. For example, factor 1 loaded on variables related to hepatic dysfunction (aspartate aminotransferase, alanine aminotransferase, and bilirubin levels) and factor 4 loaded on variables related to coagulopathy (albumin level, international normalized ratio, and hemoglobin level) (eFigure 3 in the Supplement). A density-based clustering algorithm, OPTICS, revealed a smooth reachability plot (eFigure 4 in the Supplement), suggesting that partitioning techniques with consensus K-means clustering are appropriate for subsequent phenotyping analyses.

**Figure 3.**
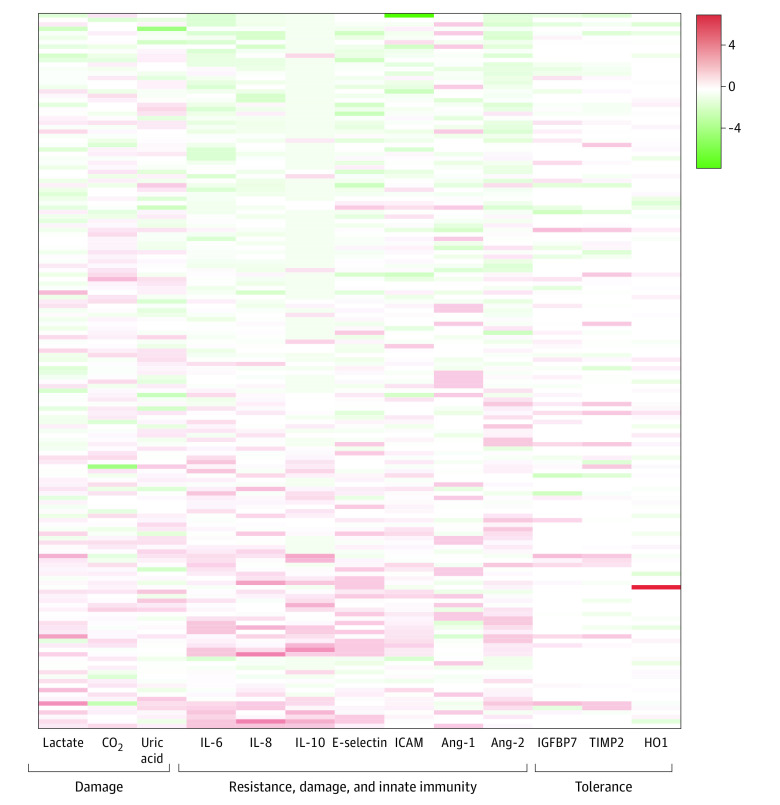
Biomarker Values Heatmap showing the log-fold change of the median biomarker value (x-axis) per patient for various markers of the host response grouped by those reflecting damage, resistance and innate immunity, and tolerance. Red represents 4 log fold greater median (4) biomarker value for that patient compared with the median of the entire study (0); green represents 4 log fold lower (−4) values of the biomarker compared with the median of the entire study. White cells are those in which the biomarker was not measured. Ang-1 indicates angiopoietin 1; Ang-2, angiopoietin 2; CO_2_, carbon dioxide; HO1, heme-oxygenase 1; IL, interleukin; IGFBP7, insulinlike growth factor binding protein 7; ICAM, intracellular adhesion molecule; and TIMP2, tissue inhibitor of metalloproteinases 2.

To understand the feasibility of remnant samples for biomarker analyses, an enriched population (n = 160) with 2 or more SOFA points was identified consecutively from the start of the study. When the data were illustrated in a heat map, we found substantial variability in the log-fold change from group median values of biomarkers of tissue damage (eg, plasma lactate, −2.3 to 3.7), resistance and innate immunity (eg, interleukin-10, −0.7 to 3 3.7), and candidate markers of organism tolerance (eg, insulinlike growth factor binding protein 7, −2.2 to 2.4) (Figure 3).

We assessed the feasibility of detecting microbiome in remnant samples. Plasma specimens contained a more bacterial DNA compared with negative control specimens (*P* < .001), with a median of 3131 bacterial gene copies/mL (interquartile range, 2012-4658 copies/mL) (eFigure 5 in the Supplement). Using 16S rRNA gene amplicon sequencing, we obtained a total of 2 882 925 amplicons in the 160 plasma specimens. The 2 most abundant taxa were both classified as members of the *Pseudomonas* genus, together composing 33% of bacterial reads.

### Financial Burden

**Figure 4.**
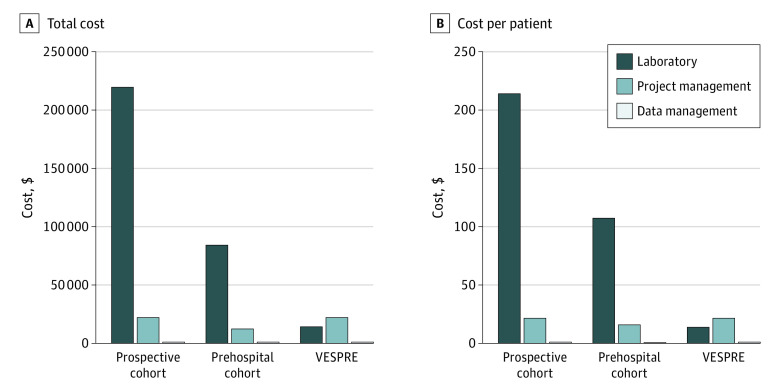
Financial Implications of Virtually Enabled Biorepository and Electronic Health Record–Embedded, Scalable Cohort for Precision Medicine (VESPRE) vs Traditional Study Designs Total cost (A) and cost per patient (B), stratified by laboratory supplies and staff, project management, and data management.

The total incremental cost beyond the existing operational infrastructure for VESPRE was $39 417.50—approximately $39 per patient (n = 1027). VESPRE costs were divided between project management ($14 880.00 [$14.48 per patient]), laboratory supplies and staff ($22 717.50 [$22.12 per patient]), and data management ($1820.00 [$1.77 per patient]). The project management cost included $13 500.00 for the electronic alert and $1380.00 for sequential updates to the alert capabilities. In comparison, a prehospital prospective cohort study (GM104022) of 787 patients completed between 2013 and 2014 cost $99 073.46 ($125.88 per patient) for coordinator-collected samples.^18^ In a hypothetical cohort study in which a coordinator core would complete the same activities as in VESPRE, including obtaining informed consent and performing biologic sampling, the budget for 1027 patients was the most expensive, costing $244 917.50 ($238.48 per patient). This cost included $14 880.00 for the electronic alert, $9300.00 for preplanning, and $196 200.00 for recruitment, screening, and enrollment (Figure 4).

## Discussion

To encourage precision medicine in acute care, we developed a tool to overcome the major barriers of scale, detail, time, and cost. We tested VESPRE, a virtually enabled biorepository system embedded in the EHR that is scalable across patients, centers, and laboratories. Use of VESPRE appears to be feasible, has proof-of-concept for disease subtyping, and is less costly for time-sensitive conditions compared with traditional cohort studies.

Precision medicine shifts from treatment of the average patient to that of the individual by targeting specific disease mechanisms. A successful precision medicine approach has been developed for melanoma, in which genetic markers, such as programmed death ligand-1, are targeted for treatment and used to predict treatment response.^3^ Precision medicine approaches such as this require substantial time, may be feasible only with slower diagnosis and treatment, and have yet to be demonstrated for acute conditions with diagnostic uncertainty, disease heterogeneity, and time-sensitive treatments. We addressed the challenges of disease acuity, timing, and cost by obtaining a waiver of informed consent and using an electronic alert embedded in the EHR to identify remnant blood and urine samples for downstream testing.

There were many lessons learned in the development and implementation of VESPRE. The first lesson relates to time. Patient enrollment can be slowed by many factors, such as diagnosis, obtaining informed consent from the patient or patient’s representative, and collection of clinical and biologic data by a study team. We captured patients with a heterogeneous syndrome (ie, sepsis) at the beginning of the care episode by embedding electronic alerts into the EHR using modified SOFA criteria. The method of enrollment eliminated the need for a dedicated study team for screening. Coupled with an institutional review board–approved waiver of informed consent and use of remnant blood and urine samples, VESPRE accomplished rapid and automated prospective enrollment at reduced time and cost. The second lesson was the creation of a biorepository. Common barriers to biorepository development include acquisition of informed consent, dedicated research blood collections, and sample processing, labeling, and storage. As with the first lesson, we overcame these challenges with a waiver of informed consent and use of remnant blood and urine samples. These steps eliminated the need for a dedicated research coordinator for sample collection, minimized risk to patients, and preempted barriers, such as poor vascular access.

VESPRE has many implications. It demonstrates a light-touch infrastructure for precision learning about biology with substantially less cost. We enrolled over 50 patients per month and collected 650 sample aliquots per month over a 20-month period at one-fifth of the cost of a traditional coordinator-led protocol. VESPRE underscores the importance of embedding screening and enrollment procedures in the EHR. By using a modified SOFA electronic alert embedded in the EHR, we captured patients with a SOFA score greater than or equal to 2, which is a key component of sepsis-3 criteria. Future iterations could use more complex screening logic, machine-learned criteria, and interoperable criteria in Fast Healthcare Interoperability Resources.

### Limitations

There are limitations to the study due to those of VESPRE. First, we only assessed the feasibility of VESPRE in one acute condition—sepsis—a focus for precision medicine. However, sepsis has heterogeneity and diagnostic uncertainty.^1^ Second, we used a modified version of diagnostic criteria in the electronic alert to facilitate screening. Although the total SOFA scores in some patients may be underestimated, clinical adjudication demonstrated the alert captures patients with a SOFA score greater than or equal to 2. Third, remnant samples may not be ideal for all proposed research tests because the biomarker measurement may not be stable for analysis after frozen. One-half of the potential remnant samples were excluded for reasons related to the integrity of the remaining sample or availability in the clinical laboratory. Fourth, we did not reproduce any existing classifiers, such as the defined clinical sepsis phenotypes.^7^ Therefore, further work is needed to ensure that data extracted by an electronic alert embedded in the EHR can be used to embed sepsis subtypes. Fifth, cost estimates were specific to this study and may not be generalizable to other institutions.

As VESPRE scales across health systems, many challenges arise. First, regulatory review may differ for each site, country, and region. Data sharing may also require distributed networks across multiple proprietary health care systems. Therefore, the suitability of VESPRE for precision medicine in acute care will vary. Second, remnant samples are not yet suitable for genetic tests. These analyses require different sample acquisition, handling, and storage conditions, and regulatory requirements—limitations that could be solved with more inclusive informed consent upon hospital admission. Third, as the biorepository expands, long-term storage strategies are required for biobanking remnant samples, which may affect cost and storage capabilities.

## Conclusions

To encourage precision medicine in acute care, we developed VESPRE, an infrastructure embedded in the EHR that creates a virtually enabled biorepository, potentially scalable across patients, centers, and laboratories. The findings suggest that VESPRE is feasible, has proof-of-concept for disease subtyping, and is less costly than traditional cohort studies for time-sensitive conditions.

## Notes

Supplement. **eTable 1.** Specimen Test Tube Details **eTable 2.** Data Missingness **eFigure 1.** Number of VESPRE Patients Categorized by Electronic Alert, Eligible, and Enrolled **eFigure 2.** Study Exclusions **eFigure 3.** Factor Analysis on VESPRE Cohort **eFigure 4.** OPTICS Plots on VESPRE Cohort **eFigure 5.** Feasibility of Microbiome Analyses in Enriched Subset
