# Supplementary material for: Feasibility of Embedding a Scalable, Virtually Enabled Biorepository in the Electronic Health Record for Precision Medicine
Source: JAMA Netw Open. 2021 Feb 22;4(2):e2037739. doi: 10.1001/jamanetworkopen.2020.37739 (PMC7900864; doi:10.1001/jamanetworkopen.2020.37739)
Supplement: Supplement. — eTable 1. Specimen Test Tube Details eTable 2. Data Missingness eFigure 1. Number of VESPRE Patients Categorized by Electronic Alert, Eligible, and Enrolled eFigure 2. Study Exclusions eFigure 3. Factor Analysis on VESPRE Cohort eFigure 4. OPTICS Plots on VESPRE Cohort eFigure 5. Feasibility of Microbiome Analyses in Enriched Subset [file jamanetwopen-e2037739-s001.pdf]

Supplementary Online Content

DeMerle KM, Kennedy JN, Peck Palmer OM, et al. Feasibility of embedding a scalable, virtually enabled biorepository in the electronic health record for precision medicine. *JAMA Netw Open*. 2021;4(2):e2037739. doi:10.1001/jamanetworkopen.2020.37739

eTable 1. Specimen Test Tube Details

eTable 2. Data Missingness

eFigure 1. Number of VESPRE Patients Categorized by Electronic Alert, Eligible, and Enrolled

eFigure 2. Study Exclusions

eFigure 3. Factor Analysis on VESPRE Cohort

eFigure 4. OPTICS Plots on VESPRE Cohort

eFigure 5. Feasibility of Microbiome Analyses in Enriched Subset

This supplementary material has been provided by the authors to give readers additional information about their work.

© 2021 DeMerle KM et al. *JAMA Network Open.*

eTable 1. Specimen test tube details

Biomarker	Test Type	Minimum Volume
**EDTA tube**		
Plasminogen activator inhibitor-1 (PAI-1)	2-Plex Luminex	50uL
Antithrombin III		
Interleukin-6 (IL-6)	7- Plex Luminex	
Interleukin-8 (IL-8)		
Interleukin-10 (IL-10)		
E-Selectin		
Angiopoietin 1 (Ang-1)		
Angiopoietin 2 (Ang-2)		
Intracellular adhesion molecule (ICAM)		
Heme oxygenase 1 (HO-1)	Human Heme Oxygenase (HO-1) ELISA	100uL
Urine		
AKI Risk Score	Nephrocheck	100uL
Tissue inhibitor of metalloproteinases 2 (TIMP2)		
Insulin-like growth factor binding protein 7 (IGFBP7)		
**Sodium Fluoride**		
Lactate	Beckman Coulter AU680 and AU5800 analyzers	200uL
**Heparin tube**		
Bicarbonate (HCO3)	Beckman Coulter AU680 and AU5800 analyzers	200uL[T]/100uL[S]
Uric Acid		
C-reactive protein (CRP)		
Procalcitonin (PCT)	BioMerieux MiniVidas Analyzer	300uL

© 2021 DeMerle KM et al. *JAMA Network Open.*

eTable 2. Data missingness (n=549)

**Variable**	**Total (n=549)**
Age	0 (0%)
Gender	0 (0%)
Race	0 (0%)
Elixhauser comorbidities	0 (0%)
Premature neutrophil count	450 (81.9%)
C-reactive protein	517 (94.2%)
Erythrocyte sedimentation rate	547 (99.6%)
White blood cell count	50 (9.1%)
Respiratory Rate	38 (6.9%)
Oxygen saturation	30 (5.5%)
Partial pressure of oxygen	475 (86.5%)
Heart rate	29 (5.3%)
Systolic blood pressure	28 (5.1%)
Troponin I	362 (65.9%)
Lactate	0 (0%)
Bicarbonate	61 (11.1%)
Creatinine	52 (9.5%)
Blood urea nitrogen	51 (9.3%)
Albumin	87 (15.8%)
Alanine transaminase	88 (16.0%)
Aspartate transaminase	88 (16.0%)
Bilirubin	88 (16.0%)
Hemoglobin	49 (8.9%)
International normalized ratio	124 (22.6%)
Platelet	51 (9.3%)
Chloride	50 (9.1%)
Glucose	46 (8.4%)
Sodium	51 (9.3%)
Glasgow Coma Scale Score	320 (58.3%)

© 2021 DeMerle KM et al. *JAMA Network Open.*

eFigure 1.

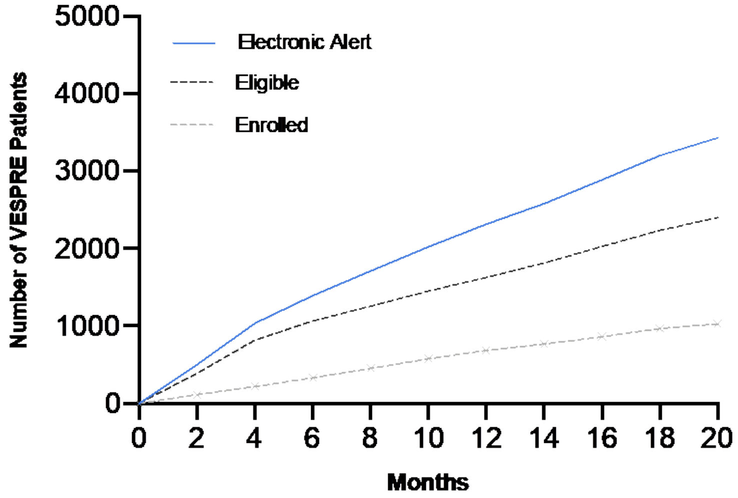

Number of VESPRE patients categorized by electronic alert (light blue), eligible (dashed dark grey) and enrolled (light grey)

© 2021 DeMerle KM et al. *JAMA Network Open.*

eFigure 2. Study exclusions

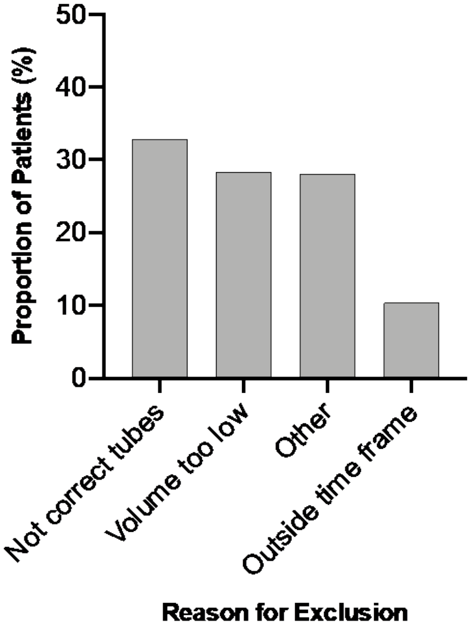

Patients categorized by incorrect blood tubes in laboratory, volume of blood remaining in specimens below minimum necessary, blood drawn after 6-hours of arrival to ED and other (unable to locate specimens, specimens discarded)

© 2021 DeMerle KM et al. *JAMA Network Open.*

eFigure 3. Factor analysis on VESPRE cohort (n=549)

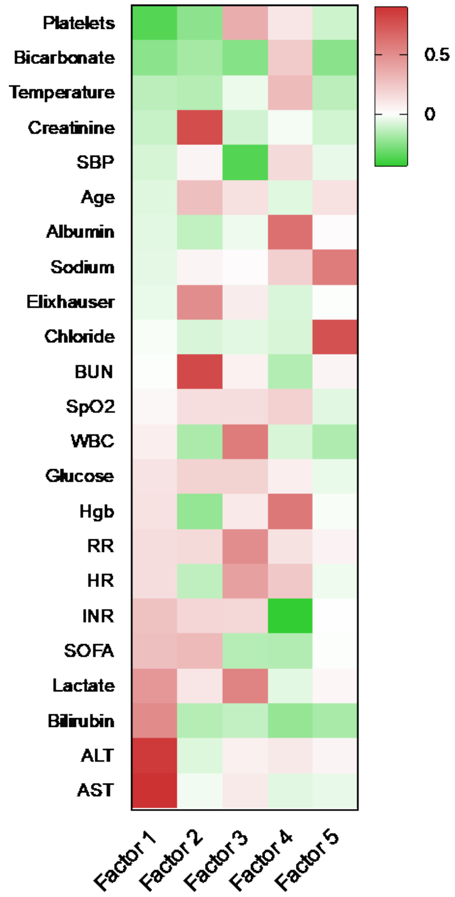

Rotated factor analysis (varimax) on 23 clinical variables revealing 5 factors with Eigenvalue >1. The gradient from green (0%) to red (100%) represents the percentage that each variable loads on the factors.

© 2021 DeMerle KM et al. *JAMA Network Open.*

eFigure 4. OPTICS plots on VESPRE cohort (n=549)

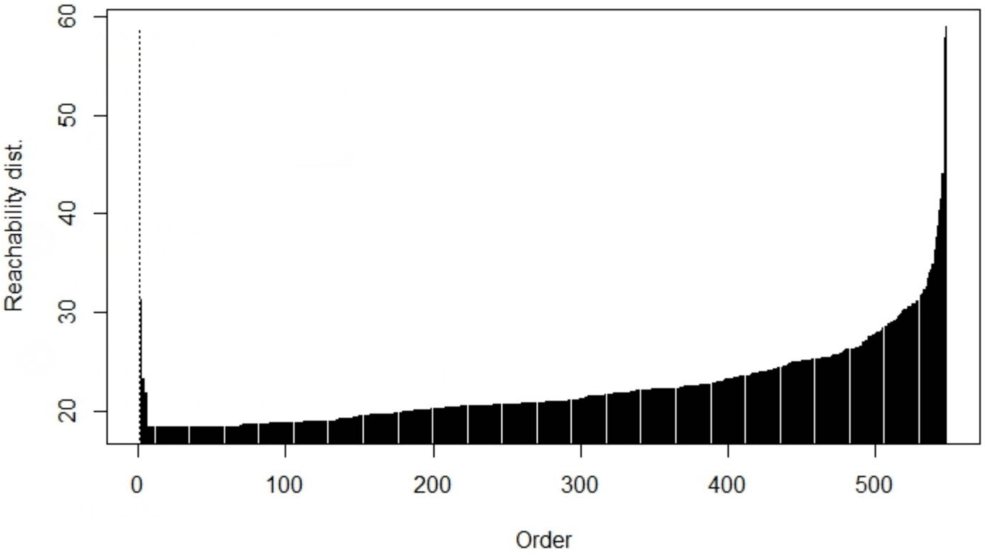

The OPTICS plot demonstrates a smooth rise in reachability distance (as opposed to well demarcated sets); therefore, partitioning methods are recommended for future machine learning methodology

© 2021 DeMerle KM et al. *JAMA Network Open.*

eFigure 5. Feasibility of microbiome analyses in enriched subset (N=160)

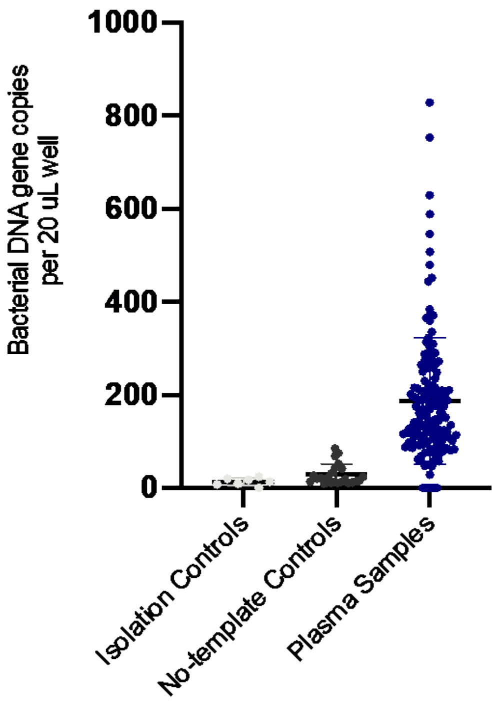

Quantification of DNA burden, quantified by bacterial DNA gene copies per 20 microliter (μL) well, of isolation controls (light grey), no-template controls (dark grey) and plasma samples (blue). Horizontal lines represent interquartile ranges (IQR)

© 2021 DeMerle KM et al. *JAMA Network Open.*
